# The Genome of the Haptophyte *Diacronema lutheri* (*Pavlova lutheri*, Pavlovales): A Model for Lipid Biosynthesis in Eukaryotic Algae

**DOI:** 10.1093/gbe/evab178

**Published:** 2021-08-03

**Authors:** Chris J Hulatt, René H Wijffels, Matthew C Posewitz

**Affiliations:** 1 Faculty of Biosciences and Aquaculture, Nord University, Mørkvedbukta Research Station, Bodø, Norway; 2 Department of Chemistry, Colorado School of Mines, Golden, Colorado, USA; 3 Bioprocess Engineering, AlgaePARC, Wageningen University and Research, The Netherlands

**Keywords:** protist, haptophyte, lipid metabolism, biotechnology, PacBio sequencing

## Abstract

Haptophytes are biogeochemically and industrially important protists with underexplored genomic diversity. We present a nuclear genome assembly for the class Pavlovales, which was assembled with PacBio long-read data into highly contiguous sequences. We sequenced strain *Diacronema lutheri* NIVA-4/92, formerly known as *Pavlova lutheri*, because it has established roles in aquaculture and has been a key organism for studying microalgal lipid biosynthesis. Our data show that *D. lutheri* has the smallest and most streamlined haptophycean genome assembled to date, with an assembly size of 43.503 Mb and 14,446 protein-coding genes. Together with its high nuclear GC content, *Diacronema* is an important genus for investigating selective pressures on haptophyte genome evolution, contrasting with the much larger and more repetitive genome of the coccolithophore *Emiliania huxleyi*. The *D. lutheri* genome will be a valuable resource for resolving the genetic basis of algal lipid biosynthesis and metabolic remodeling that takes place during adaptation and stress response in natural and engineered environments.

Significance

Haptophytes are evolutionarily significant protists, yet they are underrepresented in genomic studies and several clades have no available genomes. We used third generation long-read sequencing to assemble a high-contiguity genome for *Diacronema lutheri* and provide an initial investigation of the nuclear genome architecture of the class Pavlovales. Our results are of value for understanding the evolution of haptophytes, algal lipid metabolism, and for strain improvement in biotechnology.

## Introduction

Haptophytes comprise a major proportion of the phytoplankton community that globally have large-scale impacts on carbon cycling and ocean biogeochemistry (Liu et al. 2009; Gutowska et al. 2017; Heureux et al. 2017). They include over 300 characterized species, with hundreds more strains detected in ocean metabarcoding studies (Kim et al. 2011; Gran‐Stadniczeñko et al. 2017). The genus *Tisochrysis and Pavlova* (*Diacronema*) are especially valuable for the aquaculture and biotechnology industries, where they supply food and essential lipids for farmed fish and shellfish (Shah et al. 2018). Further developments in large-scale microalgae cultivation could expand the production of sustainable foods, oils, and plastic replacement materials in the future (Cottrell et al. 2020; Naduthodi et al. 2021).

Despite their impact and their intriguing evolutionary history, high-quality haptophyte genome sequences remain scarce, and the available data do not reflect their diversity or new discoveries (Burki et al. 2012; Sibbald and Archibald 2017; Kawachi et al. 2021). To bridge this knowledge gap, we assembled and annotated a high-quality genome for *Diacronema lutheri* (*Pavlova lutheri*), for the purpose of understanding its architecture, sequence evolution, and capacity to synthesize diverse natural products, particularly lipids.

The Pavlovales, which include the genus *Pavlova*, *Diacronema*, *Rebecca*, and *Exanthemachrysis*, invariably occupy the outer branches of haptophyte phylogenies, some considerable evolutionary distance from the coccolithophores, *Chrysochromulina and Phaeocystis* clades (Egge et al. 2015; Edvardsen et al. 2016; Song et al. 2021). *Pavlova* sp. cells are about five microns in size with a short haptonema, their swimming motion driven by two flagella of slightly unequal length. They are unusual among microalgae as a combined source of eicosapentaenoic acid (20:5*n*−3) and docosahexaenoic acid (22:6*n*−3) that are integrated into different types of structural and storage lipids (Meireles et al. 2003). *Pavlova* sp. also synthesize additional betaine lipids including 1,2-diacylglyceryl-3-*O*-carboxyhydroxymethylcholine (DGCC) and 1,2-diaclyglyceryl-3-*O-*2′-(hydroxmethyl)-(N, N, N-trimethyl)-β-alanine (DGTA), plus some unusual dihydroxylated sterols (pavlovols) that are unique to the class (Volkman 2016; Li-Beisson et al. 2019; Marcellin-Gros et al. 2020).

A few studies have amplified and sequenced individual *Pavlova* sp. genes (Tonon et al. 2003; Robert et al. 2009), but the majority of the nuclear genome remains unexplored. Nosenko et al. (2007) used pulsed-field electrophoresis to estimate a modest genome size of 20.7 and 28.7 Mb for *Diacronema* sp. and *Pavlova gyrans*, respectively, so we expected a comparable result. Here, we assembled the genome of strain “*Pavlova* sp. NIVA-4/92,” which we identify as *D. lutheri* (synonymous with *P. lutheri*) based on its mitochondrial, plastid, and 18S sequences (Hulatt et al. 2020). We primarily used long PacBio reads at high coverage with the aim to comprehensively unravel key biosynthetic pathways, resolve evolutionary relationships among genes, and determine mechanisms controlling triacylglycerol biosynthesis and adaptive lipid remodeling (Cañavate et al. 2017; Wei et al. 2017). Our data will also be valuable for applied studies of genome-informed strain improvement and models of cell metabolic flux.

## Results and Discussion

### Genome Size and Quality

The *D. lutheri* nuclear genome assembly is 43,502,671 bp in size and contains 14,446 annotated protein-coding genes, making it the most compact among sequenced haptophytes (table 1). The assembly consists of 103 contigs, with approximately half of the total sequence length contained in 16 contigs. These high-contiguity sequences reflect the advances in long-read sequencing technology compared with earlier Illumina and 454-based methods. The theoretical coverage of the PacBio reads is ×368, and in practice the nuclear genome coverage is most commonly about ×315. The genome size is 1.5–2 times larger than that previously predicted for other related Pavlovales, but the contig lengths are within the expected chromosome size range of 0.18–4 Mb (Nosenko et al. 2007). Analysis of the genome sequence with BUSCO v.4 identified 80.8% of core eukaryotic genes were complete and only 0.4% of these were duplicated (table 1). Compared with the other haptophyte assemblies, these scores support a rather complete genome with minimal sequence duplication.

**Table 1.** Comparison of Four Published Haptophyte Genomes with *Diacronema lutheri* NIVA-4/92.

	*Diacronema lutheri*	*Tisochrysis lutea*	*Chrysochromulina tobin*	*Chrysochromulina parva*	*Emiliania huxleyi*
	JAGTXO010000000	TisoV1	GCA_001275005.1	GCA_002887195.1	GCF_000372725.1
Assembly length (contigs) (Mb)	43.503	57.719	59.073	65.765	155.931
Total number contigs	103	9,930	3,412	8,362	16,921
Scaffolds	—	7,695	—	—	7,795
Contig N50	16	1,970	798	1,243	1,314
Contig L50 (kb)	852.26	8.07	24.11	16.05	29.72
Longest contig	3.042 Mb	726.925 kb	121.428 kb	101.752 kb	299.609 kb
GC content (± contig)	73.25%	58.67%	63.37%	63.58%	65.67%
	±1.34%	±2.95%	±2.74%	±3.99%	±4.13%
Method	PacBio + Illumina	Illumina	Illumina + 454	Illumina	Sanger
Complete (%)	80.80	68.30	62.00	72.90	51.80
Complete, single copy (%)	80.40	65.90	61.60	72.50	37.30
Complete, duplicated (%)	0.40	2.40	0.40	0.40	14.50
Fragmented (%)	6.30	11.40	7.50	7.10	16.10
Missing	12.90%	20.30%	30.50%	20.00%	32.10%
Genes^a^	14,446	20,582	16,777	28,138	30,569
Annotation method	BRAKER2	MAKER2	MAKER2	MAKER2	JGI Annotation Pipeline
Reference	This study	Carrier et al. (2018)	Hovde et al. (2015)	Hovde et al. (2019)	Read et al. (2013)

Note.—Assembly statistics are based on contigs for comparability. BUSCO v.4 was run on the genome sequences with the “eukaryote_odb10” data set.

a Structural annotation of genes are as reported in the corresponding manuscripts, which were annotated with different methods.

The *D. lutheri* nuclear genome assembly has a high 73.25% GC content that is reflected in the raw PacBio subreads and in the Illumina reads (supplementary figs. 1 and 2, Supplementary Material online). It surpasses that of the coccolithophore *Emiliania huxleyi* (65.67%) and is among the highest observed in eukaryotic cells. Understanding the selective mechanisms driving this elevated GC skew might help explain patterns in haptophyte evolution, and could also support detection of cryptic picoplanktonic haptophytes in metagenomes (Liu et al. 2009; Edvardsen et al. 2016).

### Repetitive Elements

Approximately 22.9% of the *D. lutheri* genome is repetitive, substantially less than the *E. huxleyi* genome, of which 64% was classified as repeats (Read et al. 2013). Long-terminal repeats (LTRs) and secondarily long-interspersed terminal repeats (LINEs) comprised the majority of the annotated repeat elements in *D. lutheri*, representing 32.7% and 3.8% of masked bases, respectively (supplementary table 1, Supplementary Material online).

### Gene Annotations

**Fig. 1.**
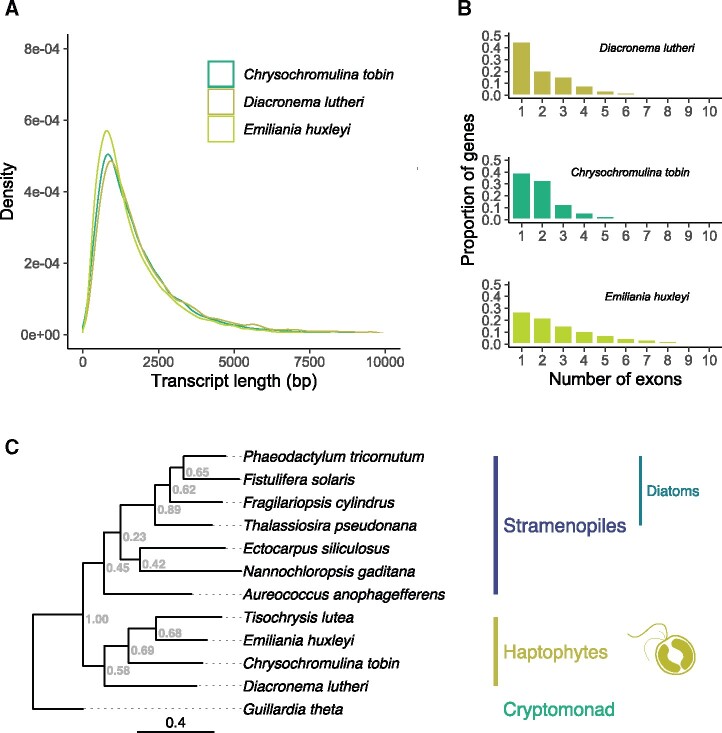
(*A*) Gene transcript length distributions for *Diacronema lutheri* annotated in this study compared with two other haptophytes, *Emiliania huxleyi and Chrysochromulina tobin*. (*B*) The number of exons per gene for the same three genomes, expressed as a proportion of the total number of annotated genes. (*C*) The species tree of 12 protists bearing red alga derived plastids, including four sequenced haptophytes, seven stramenopiles, and one cryptomonad. The tree is derived from 1,152 orthogroups with at least one gene copy from each species. Branch lengths represent substitutions per site and support values are derived from the STAG algorithm implemented in OrthoFinder2.

The total length of protein-coding nucleotides is 26.62 Mb which represents 61.2% of the genome. The gene length and exon counts of *D. lutheri* were compared with the structural annotations of *E. huxleyi and Chrysochromulina tobin* (fig. 1*A and B*). Single-exon genes account for 45% of the *D. lutheri* coding sequences, with fewer genes containing a single intron (21%) or multiple introns (34%). The *C. tobin* genome encodes fewer single-exon genes whereas *E. huxleyi*, with the largest sequenced genome, encodes only 27% single-exon genes, with 50% of genes containing two or more introns. Such variation raises questions on patterns of genome-wide intron gain and loss in haptophytes, and the extent to which posttranscriptional regulation by alternative splicing is prevalent across different clades.

In total 9,498 of the 14,446 protein-coding genes received at least one gene ontology (GO) identification. An initial survey of genes related to lipid metabolism identified 25 proteins with annotated desaturase activity, six with elongase activity, and 54 with acyltransferase functions. Thirty-seven tRNAs decoding the standard 20 amino acids were annotated by tRNAscan-SE, and an additional 42 ncRNAs were annotated by Infernal/Rfam.

### Gene Orthology Comparative Genomics

The amino acid sequences from the whole genome of *D. lutheri* were compared with those of three available haptophyte data sets, and with eight further data sets from more distantly related red-plastid bearing species, using OrthoFinder2 (Emms & Kelly 2019). From the four haptophyte sequence sets and a total of 89,703 genes, 749 orthogroups contained single-copy genes, whereas 3,438 orthogroups contained at least one gene ortholog from each species. For the expanded 12 species set of amino acid sequences, a total of 213,864 genes were distributed among 25,757 orthogroups, of which 1,152 orthogroups were common to all 12 genomes, but only five were single-copy orthologs found in all organisms. Figure 1*C* displays the species-tree using the amino acid sequences from the 12 genomes.

## Conclusions

We assembled a new haptophyte genome for the class Pavlovales with long PacBio reads to build high contiguity sequences. The genome size, gene, and GC content of *D. lutheri* places the Pavlovales as an important clade for understanding selective processes and genome streamlining among ecologically and biogeochemically important haptophytes. Our results will be more fully exploited through investigation of lipid metabolism, metabolic modeling, and strain improvement for industrial bioprocesses.

## Materials and Methods

### Cell Culture Preparation

Strain “*Pavlova* sp. NIVA-4/92” was obtained from the Norwegian Culture Collection of Algae (NORCCA). This species reportedly originates from Oslofjord, Norway, and has been held in culture since 1989. Cells were cultivated in f/2 medium (Guillard and Ryther 1962) using 0.2 µm filtered and autoclaved seawater containing the antibiotics ampicillin, kanamycin, and streptomycin. Clonal cultures were obtained by cell-sorting with an Astrios EQ flow cytometer (University of Colorado Cancer Center, Denver, CO). Cell cultures were prepared in 500 ml bioreactors bubbled with filtered air containing 1% CO_2_. In the late exponential phase, the cells were collected and pelleted by centrifugation, then flash-frozen in liquid N_2_ and stored at −80 °C.

### DNA Sequencing

High molecular weight DNA was extracted from cells and the fragments were size selected at over 30 kb by Arizona Genomics Institute (Tucson, AZ). After SMRTbell library preparation, sequencing was performed on a PacBio Sequel system using three 1M SMRT cells with v2.1 chemistry and 10 h movies. The raw data were processed with the command line tools from SMRTLink v.5.1 and the total yield was 993,273 subreads (16.6 Gb) with N50 length 23.458 kb. The longest subread was 92.675 kb. The GC content of subreads initially indicated a nuclear genome with a high GC content of ∼70% (supplementary fig. 1, Supplementary Material online). Short-read sequencing was performed with an Illumina MiSeq producing 250 bp paired-end reads on one v.2 flow cell, yielding 6.55 Gb of reads. The Illumina sequences were trimmed with Trimmomatic (Bolger et al. 2014) and quality checked by FastQC (Babraham Bioinformatics).

### RNA Samples and Sequencing

Three independent RNA sequence libraries were obtained from two different experiments. Experiment 1 comprised a set of pooled Erlenmeyer flask cultures exposed to six different stress conditions (control, low-nutrient, low-temperature, low salinity, darkness, high light) to express the maximum number of genes. Experiment 2 was a bioreactor study from which two representative RNA samples (one control and one phosphorus-limited treatment) were selected. In each case, RNA was extracted from cell pellets using Trizol reagent and chloroform, followed by an RNA Clean & Concentrate mini-column preparation (Zymo Research, Irvine, CA). Illumina sequencing was performed by Novogene (Beijing, China) Ltd, yielding 21.4, 28.4 and 20.0 million cleaned and trimmed 150 bp paired-end strand-specific reads (fragments) from each of the three libraries, respectively.

### Genome Assembly and Polishing

The PacBio data were used for genome assembly, whereas the Illumina sequences were used only for polishing the assembled contigs. PacBio subreads were assembled with CANU v.1.7 and the options “minReadLength = 3,000” “corOutCoverage = 100” “correctedErrorRate = 0.04” (Koren et al. 2017). The organelle sequences were identified and extracted from the whole genome assembly and finished separately (Hulatt et al. 2020). To minimize errors in the assembled sequences the contigs were polished three times with the PacBio command line tools in SMRTLink v.5.1 (Pacific Biosciences, Menlo Park, CA), where the raw subreads were aligned to the contigs with BLASR (Chaisson and Tesler 2012) and polished with the ARROW hidden Markov model to high consensus accuracy. Next, the 250-bp PE Illumina reads were used for final polishing to eliminate potential remaining small indels and single base errors. To do this the Illumina reads were aligned to the contigs using BWA-MEM (Li 2013), polished using Pilon for three rounds (Walker et al. 2014), and subsequently polished using FreeBayes (Garrison and Marth 2012) for three rounds. Genome coverage by the Illumina data was approximately 150-fold on an average.

### Genome Curation

The initial assembly contained 136 contigs with a total length of 45.09 MB. To objectively identify and remove potentially erroneous, short or duplicated sequences derived from low-abundance or contaminant reads, the PurgeHaplotigs pipeline was applied (Roach et al. 2018). Raw PacBio subreads were mapped to the genome using Minimap2 with the options “-ax map-pb” (Li 2018) and spurious contigs were removed by defining lower, mid, and upper coverage limits. This process eliminated 33 relatively short sequences of total length 1.6 Mb and average length 48 kb, or about twice the N50 read length. The curated assembly was finally assessed for possible remaining contamination using BLAST against the “nt” database followed by manual inspection of top hits, but no further contigs were removed.

### Genome Quality Assessment

Genome quality was monitored through the assembly and curation process using BUSCO (Seppey et al. 2019) and results presented in this manuscript are from BUSCO v.4.0.2 and the “eukaryote_odb10” collection of 255 conserved core eukaryotic genes. For comparative purposes, BUSCO was run on the genome sequences of *D. lutheri* and four other haptophyte assemblies with the optimized “–long” two-pass option and otherwise default BLAST settings.

### Structural Annotation of Genes

To characterize repetitive regions a custom repeat library was constructed de novo using REPEATMODELER with all contigs over 100 kb (Smit et al. 2015a). The genome sequence was soft-masked using REPEATMASKER with the option “-xsmall” (Smit et al. 2015b). Gene structural annotation was subsequently performed with the BRAKER2 pipeline using RNA-seq evidence combined with AUGUSTUS and GENEMARK-ES for gene prediction (Bruna et al. 2021). The three RNA-seq libraries were aligned individually to the genome using STAR v.2.7.3a (Dobin et al. 2013) (supplementary table 2, Supplementary Material online) and the braker.pl pipeline was provided the RNA-seq read alignments and run with the “–softmasking” option.

### Functional Annotation of Genes

To assign functions to the protein-coding sequences three different methods were used in parallel and the consensus results were collected: 1) INTERPROSCAN-5 was used to search for conserved protein signatures (Jones et al. 2014), 2) Protein sequences were searched with BlastP against the curated SwissProt database (Boeckmann et al. 2003), and 3) EGGNOG-MAPPER was used for orthology assignment, running emapper.py with DIAMOND alignment (Huerta-Cepas et al. 2017). Transfer RNAs were annotated with tRNAscan-SE v.2.0.7 with recommended settings for eukaryote genome annotation (Chan and Lowe 2019). Noncoding RNAs were annotated with INFERNAL (Nawrocki and Eddy 2013) and the Rfam library of covariance models (Kalvari et al. 2018).

### Genome Sequences and Gene Orthology

Three haptophyte genome data sets were obtained from NCBI GenBank (*E. huxleyi* assembly GCA_000372725.1; *C. tobin* assembly GCA_001275005.1; *Chrysochromulina parva* assembly GCA_002887195.1) and one data set for *Tisochrysis lutea* was obtained from SEANOE (assembly v1; https://www.seanoe.org/data/00361/47171/; last accessed April 6, 2021; doi:10.17882/47171). Amino acid coding sequences for a further eight species were obtained from NCBI GenBank (*Thalassiosira pseudonana* CCMP1335 GCA_000149405.2; *Phaeodactylum tricornutum*GCA_000150955.2; *Aureococcus anophagefferens*GCA_000186865.1; *Ectocarpus siliculosus*GCA_000310025.1; *Guillardia theta* CCMP2712 GCA_000315625.1; *Nannochloropsis gaditana* B-31 GCA_000569095.1; *Fragilariopsis cylindrus*GCA_001750085.1; *Fistulifera solaris*GCA_002217885.1).

## Supplementary Material

Supplementary data are available at *Genome Biology and Evolution* online.

## Supplementary Material

evab178_Supplementary_Data
