# Supplementary material for: The Genome of the Haptophyte Diacronema lutheri (Pavlova lutheri, Pavlovales): A Model for Lipid Biosynthesis in Eukaryotic Algae
Source: Genome Biol Evol. 2021 Aug 3;13(8):evab178. doi: 10.1093/gbe/evab178 (PMC8379373; doi:10.1093/gbe/evab178)
Supplement: evab178_Supplementary_Data [file evab178_supplementary_data.pdf]

*Supplementary Data*

# The genome of the haptophyte *Diacronema lutheri* (*Pavlova lutheri*, Pavlovales): A model for lipid biosynthesis in eukaryotic algae

Chris J. Hulatt[1,2]*, René H. Wijffels[1,3], Matthew C. Posewitz[2]

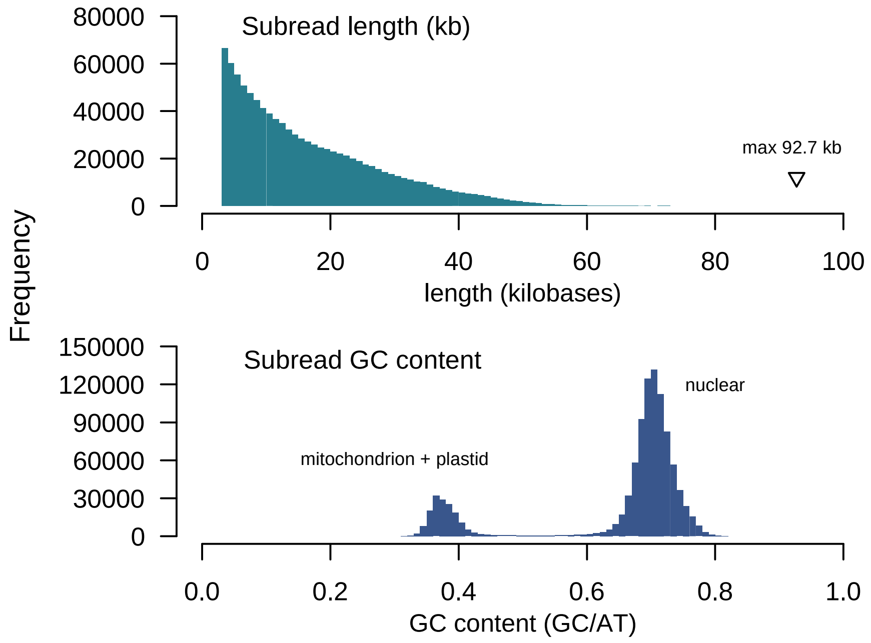


**Supplementary Fig 1.** PacBio subreads longer than 3 kb used for genome assembly with CANU. Upper panel shows the subread length distribution. Lower panel shows the subread GC content distribution with the organelle and nuclear genome reads indicated.

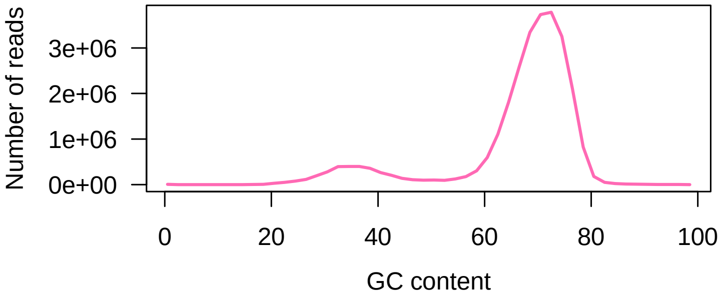


**Supplementary Fig 2.** The GC content distribution of the 250 bp Illumina MiSeq reads used for polishing the assembled genome.

**Supplementary Table 1.** Repetitive elements in the genome of *Pavlova lutheri*.

| | |
|---|---|
| Bases masked | 9,973,521 bp |
| % Genome masked | 22.93% |
| SINE short interspersed nuclear element | 118 bp |
| LINE long interspersed terminal repeat | 377360 bp |
| LTR long terminal repeats | 3263503 bp |
| DNA elements | 64612 bp |

**Supplementary Table 2.** Read alignment summary of three 150 bp Illumina RNA-seq libraries mapped to the nuclear genome. Unmapped reads include organelle sequences. The alignment files were passed to the BRAKER2 pipeline for structural annotation of CDS.

| **Sample ID** | **Number of fragments** | **% Uniquely mapped** | **% Multi mapped** | **% Unmapped** |
|---|---|---|---|---|
| Pooled | 21,405,601 | 89.73% | 5.96% | 4.31% |
| Chemostat 1B | 28,381,363 | 91.65% | 6.18% | 2.17% |
| Chemostat 2B | 20,032,843 | 91.54% | 5.64% | 2.81% |
